# Fungus Tumour of the Bones

**Published:** 1847-03

**Authors:** 


*Fungus tumour of the Bones* By Professor Roux.—A man aged thirty-eight, of a strong constitution, was lately admitted into the Hotel Dieu, for the treatment of a tumour, which had formed three months since in the right knee. At the beginning of the disease he made, on two occasions, a violent effort with the limb, and each time a very painful sense of distention was experienced in the joint. On examination two tumours were distinguished, one on either side of the tuberosity of the tibia ; pressure reduced them to a certain degree, and at all times they were the seat of pulsations synchronous to the pulse. The skin had retained its natural colour, and the patient complained only of weakness of the extremity.

After relating the case, the Professor proceeded to state that this tumour should be considered as an aneurismal affection, occupying the spongy texture of the head of the tibia. "This," continued M. Roux, "is an uncommon disease, or at least one which authors have seldom described. Fifteen or eighteen cases at most are on record. For my part, during an experience of thirty years of hospital practice, I have met only with three or four, the last of which I observed not more than two years ago; a complete cure was obtained by ligature of the femoral artery."

These tumours are almost constantly found in the bones of the extremities, particularly the lower, and in the parts which contain a greater quantity of spongy texture. The tibia is most usually occupied. An external injury, a blow, or a sprain, seems to have produced it in every instance, although it certainly is not easy to understand what influence external causes can have on the aneurismal developement of the arterial capillaries of a bone ; still the fact is one confirmed by too frequent observations to admit of doubt. In a case related by M. Lallemand, a rheumatic or gouty predisposition seemed to have caused the malady ; and in a most interesting observation of Scarpa, the same predisposition is stated to have existed. In the present case it was after a violent effort to raise a carriage that the patient first experienced pain in the part, and it was also an effort of the same kind which produced the disorder in the patient he observed about two years ago. In one patient you may remark that the internal layers of the bone have been gradually absorbed, and that the fungous areolar growth lies immediately under the skin, forming irregularities in the outward aspect of the limb. Under the name of "angiectasy" we comprise only the diseases of any part of the vascular system attended with dilatation of the vessels, and this great class may be subdivided according to the seat of the alteration in large vessels or in the capillaries. To the former belongs the history of aneurism ; to the latter, the tumours of the nature of that which has occasioned this lecture. Fungous tumours of the capillary vessels may be met with in the soft textures or in the bones, and when they occupy the latter seat, they are not only remarkable on account of their unfrequency, but also by some other circumstances, which we will briefly enumerate. In the first place we observe that sanguineous tumours of the soft parts are generally prognosticated by the presence of nævi, whereas in the bones we have no reason to suppose that the developement of the disease is anything but accidental. A proof that a congenital disposition has little or nothing to do with the aneurismal dilatation of the osseous capillaries may be found in the fact that in most cases external injuries have been the producing causes. Another material difference resides in the fact, that these tumours occupy indiscriminately the arterial or venous capillaries in the soft parts, and exclusively the arterial system of bones. A cancerous cement not unfrequently is superadded to the vascular transformation, and when this unfortunate complication is observed, the fatal result of organic disease are produced even before abundant hemorrhage could supervene. You are aware gentlemen, that, for the treatment of these erectile tumours of the soft parts, numerous methods are employed, and particularly within the last few years the progress of the art of curing has been, in this respect, truly remarkable. We recollect that twenty-five years ago, amputation was freqnently considered as the only resource in cases which now-a-days would obtain relief by a great many plans, differing with circumstances, and with surgeons. Ligature, pressure, cauterization, puncture—simple, or combined with the application of heat or of electricity: such are the principal methods of treatment which would be resorted to.

As to fungous tumours of the bones, we know only of three operations by which a cure can be obtained:—ligature of the main artery of the limb, amputation of the extremity, and, in some few instances, resection of the portion of bone occupied by the malady. Ligature we prefer, in the present instance, for reasons which we will expose in another lecture.—*London Medical Times.*

